# Radial ESWT combined with a specific rehabilitation program (rESWT+RP) is more effective than sham rESWT+RP for acute hamstring muscle complex injury type 3b: a randomized, controlled trial

**DOI:** 10.1093/bmb/ldaf009

**Published:** 2025-09-02

**Authors:** Javier Crupnik, Santiago Silveti, Natalia Wajnstein, Alejandro Rolon, Tobias Wuerfel, Peter Stiller, Antoni Morral, John P Furia, Nicola Maffulli, Christoph Schmitz

**Affiliations:** KinEf Sports Physiotherapy Center, Av. Cabildo 808 2°C, C1026 Cdad. Autónoma de Buenos Aires, Argentina; Physiotherapy School Medicine and Health Sciences Faculty, Universidad Abierta Interamericana, Chacabuco 90, C1069 Cdad. Autónoma de Buenos Aires, Argentina; KinEf Sports Physiotherapy Center, Av. Cabildo 808 2°C, C1026 Cdad. Autónoma de Buenos Aires, Argentina; KinEf Sports Physiotherapy Center, Av. Cabildo 808 2°C, C1026 Cdad. Autónoma de Buenos Aires, Argentina; Imaxe Diagnostic Imaging Center, Av. Córdoba 2340, C1120 Cdad. Autónoma de Buenos Aires, Argentina; Extracorporeal Shock Wave Research Unit, Chair of Neuroanatomy, Institute of Anatomy, Faculty of Medicine, Ludwig Maximilian University Munich, Pettenkoferstr. 11, 80336 Munich, Germany; Department of Sports Orthopaedics, Technical University of Munich, Ismaninger Str. 22, 81675 Munich, Germany; Department of Sports Medicine, Medworks Clinic, Neuburger Str. 11, 86167 Augsburg, Germany; Facultat de Ciències de la Salut Blanquerna, Universitat Ramon Llull, C/ de Padilla, 326, 08025 Barcelona, Spain; SUN Orthopedics of Evangelical Community Hospital, 210 JPM Rd, STE 300, Lewisburg, PA 17837, United States; Department of Trauma and Orthopaedic Surgery, Faculty of Medicine and Psychology, University La Sapienza, Azienda Ospedaliera Sant'Andrea, Via Giorgio Nicola Papanicolau s.n.c., 00189 Roma, Italy; School of Pharmacy and Bioengineering Keele University Faculty of Medicine, Hornbeam Building, Keele ST5 5BG, United Kingdom; Centre for Sports and Exercise Medicine, Barts and the London School of Medicine and Dentistry, Mile End Hospital, Queen Mary University of London, Mile End Road, London E1 4NS, United Kingdom; Extracorporeal Shock Wave Research Unit, Chair of Neuroanatomy, Institute of Anatomy, Faculty of Medicine, Ludwig Maximilian University Munich, Pettenkoferstr. 11, 80336 Munich, Germany

**Keywords:** extracorporeal shock wave therapy, ESWT, hamstring, radial extracorporeal shock wave therapy, rESWT, randomized controlled trial, RCT, rehabilitation, structural muscle injury

## Abstract

**Introduction:**

Acute type 3b injuries of the hamstring muscle complex (HMC) are prevalent in sports, often lead to prolonged recovery, and demonstrate a high recurrence. Conservative rehabilitation is standard, and adjunct therapies such as radial extracorporeal shock wave therapy (rESWT) may offer additional benefits.

**Sources of data:**

This randomized controlled trial, with blinding of patients and assessors, included 36 semi-professional athletes with ultrasound-confirmed acute type 3b HMC injuries. Participants received either real or sham rESWT in combination with an 8-week structured rehabilitation program. The primary outcome was time to return to sport; secondary outcomes included post-treatment muscle strength, patient satisfaction, and re-injury rate.

**Areas of agreement:**

Progressive rehabilitation is effective for muscle injuries. rESWT is a safe, non-invasive modality with high therapeutic potential in musculoskeletal conditions.

**Areas of controversy:**

Questions remain regarding the ideal rESWT protocol for acute muscle injuries, including optimal dosing, frequency, and timing relative to injury onset.

**Growing points:**

The addition of rESWT resulted in a statistically significant reduction in return-to-sport time [25.4 ± 3.5 (mean ± SD) days with rESWT vs 28.3 ± 4.5 days with sham rESWT; *P* = .037]. In elite and semi-professional athletes, even modest reductions in downtime can carry meaningful performance and economic benefits. Furthermore, only the rESWT group avoided strength deficits in the previously injured leg, suggesting improved functional recovery.

**Areas timely for developing research:**

Future studies should explore the comparative effectiveness of individualized versus standardized rESWT protocols and investigate its broader applicability across sports disciplines and levels of play.

## Introduction

Acute hamstring muscle complex (HMC) injuries are among the most frequent sports injuries overall, and represent the most common type of sports-related injury in soccer [1]. Recurrent HMC injuries are also prevalent, with recurrence rates reaching up to 25% within one year [2].

The Munich Consensus Statement classifies muscle injuries into non-structural (types 1 and 2) and structural (types 3 and 4) [3]. Structural injuries are further subdivided into partial lesions of minor extent (<5 mm, type 3a), partial lesions of greater extent (>5 mm, type 3b), and complete muscle tears or tendinous avulsions (type 4) [4].

Treatment of HMC injuries can be complex. While type 4 injuries often necessitate surgical intervention [4], partial lesions (types 3a and 3b) are typically managed conservatively. The most effective treatment approach for type 3a and 3b injuries is generally a progressive physical therapy program [5–7]. Alternative options, such as biologic agent injection therapies, have produced mixed results [8].

Recovery from type 3a and 3b injuries using conventional physical therapy may be prolonged, often requiring up to 6 weeks before return to sport [3]. Additionally, the high rate of re-injury following acute HMC injuries highlights the potential limitations of standard rehabilitation protocols. As a result, there is growing interest in developing novel treatment strategies, particularly for acute HMC injury type 3b.

In the last three decades, extracorporeal shock wave therapy (ESWT) has emerged as a safe and effective non-invasive treatment modality for a variety of musculoskeletal disorders [9–11]. Basic science research and animal studies indicate that ESWT may accelerate muscle healing [12–14], a finding supported by several case reports and case series [15, 16]. However, randomized controlled trials (RCTs) evaluating the efficacy and safety of ESWT specifically for acute HMC injury type 3b have not yet been conducted.

This study aimed to evaluate the hypothesis that the combination of radial ESWT (rESWT) and a structured rehabilitation program (RP) is both effective and safe for treating acute HMC injury type 3b in athletes, and that it is superior to the combination of sham-rESWT and RP. Given the demonstrated efficacy and safety of rESWT in treating chronic proximal hamstring tendinopathy in professional athletes [17], its use was incorporated into the current study.

Previous research conducted by our group [15] has demonstrated that administering rESWT for muscle injuries either daily or every other day can significantly reduce lay-off times in professional athletes—by 54% and 58% (median/mean) for type 1a injuries (fatigue-induced muscle disorders/muscular tightening/hypertonicity), 50% and 55% for type 2b injuries (muscle-related neuromuscular disorders/muscle strain injury) and 8% and 21% for type 3a injuries—compared with data reported for 31 European professional male soccer teams [18]. This frequency of treatment sessions is notably higher than the standard interval of 7 days (one treatment session per week) commonly used in most studies investigating ESWT for musculoskeletal conditions [9]. Since daily rESWT was not feasible in the context of this study, we implemented a protocol of three rESWT sessions per week, which closely approximates an every-other-day schedule.

## Methods

### Study design

This study was designed as a randomized, controlled, superiority trial in which patients were assigned to either rESWT + RP or sham rESWT + RP. Blinding was maintained for the assessors (SS and NW) and the statistician (CS). The trial protocol was published [19], and the study was prospectively registered on 22 March 2018 (ClinicalTrials.gov ID NCT03473899). All patients—semi-professional athletes receiving fees or university scholarships—were recruited from a sports club in the Province of Buenos Aires (Argentina) between May 2018 and December 2020. Club officials were instructed to ensure that any athlete experiencing sudden, sharp pain in the posterior thigh during training or competition ceased activity immediately and reported to the clinic of the Principal Investigator (JC) (hereafter: PI). These individuals were then assessed for eligibility based on the trial’s inclusion criteria. Assessments were conducted at baseline, on each day that rESWT or sham rESWT was administered, and subsequently every second day until return to sport was achieved.

The randomization scheme was generated by a medical assistant at the PI’s clinic using the website www.randomization.com and was not disclosed to anyone else. Forty patients were randomized into five blocks. Allocation was implemented using opaque, sealed envelopes labeled according to the randomization schedule. Group assignments remained concealed from both patients and assessors until all recruitment was complete and irreversible. Following completion of the baseline assessment, the PI opened the next sealed envelope to determine the patient’s group allocation, marking the formal entry of the patient into the trial. No patient was replaced after randomization.

The PI, who administered both rESWT and sham rESWT, was not blinded, as experienced rESWT practitioners can typically distinguish between real and sham treatments based on patient reactions—since effective rESWT generally elicits some degree of discomfort, consistent with its mechanism of action [20]. To mitigate potential bias, a strict and standardized protocol was implemented to ensure uniform interaction between the PI and all patients, regardless of treatment group [20, 21].

Blinding of patients in RCTs involving either rESWT or sham rESWT is a standard practice [22–24]. This approach necessitates that patients have not previously undergone rESWT treatment, as prior experience could allow them to discern their group allocation based on the discomfort typically associated with active rESWT. The conditions at the sports club in the Province of Buenos Aires (Argentina), where this study was conducted between May 2018 and December 2020, were particularly well-suited for this requirement, as rESWT—and ESWT more broadly—was not a commonly used treatment modality in Argentina during that period.

### Patients, therapists, centers

Adults aged 18 to 35 years (both female and male) with a clinical and ultrasonographic diagnosis of acute HMC injury type 3b were eligible for inclusion.

The inclusion criteria were: no surgical intervention required, the patient’s willingness to participate in the trial, written informed consent personally signed and dated by the patient, and absence of contraindications to rESWT.

Exclusion criteria included: individuals under 18 or over 35 years of age; patients with a clinical and ultrasonographic diagnosis of acute HMC injury type 3b who had sustained the injury >7 days prior to potential enrollment; patients diagnosed with acute HMC injury type 3a or type 4; those with bilateral acute HMC injuries (types 3a, 3b, or 4); patients with confirmed or suspected HMC injuries (types 3a, 3b, or 4) affecting the same leg within 6 months prior to potential enrollment; injuries caused by external trauma to the posterior thigh (Category B3); surgery on the affected leg within the preceding year; or acute or chronic lumbar pathology (as in some patients thigh pain may originate from spinal conditions [25]). Additional exclusion criteria were: unwillingness to participate in the trial; failure to provide personally signed and dated written informed consent; and any contraindications to rESWT, including pregnancy, blood-clotting disorders (such as local thrombosis), use of oral anticoagulants, local bacterial or viral infections/inflammations, local tumors or local corticosteroid injections within 6 weeks prior to the first rESWT session (if applicable).

The sports club in the Province of Buenos Aires (Argentina), where this study was conducted, does not routinely perform strength assessments of the athletes’ legs. Consequently, no baseline strength measurements—particularly of the dominant leg in soccer players—were available.

### Intervention

All patients participated in a specific RP lasting 8 weeks, regardless of their individual time to return to sport, in accordance with established protocols [26]. This RP was developed based on literature recommendations [27–29]. The primary objective of the RP was to help patients regain functional, neuromuscular and biomechanical capacities tailored to the demands of their respective sports while minimizing the risk of re-injury. The program guided patients through a structured progression of low-risk to high-demand movements using a systematic, phase-based approach. This included an acute phase, subacute/regeneration phase and functional phase, with each phase contingent upon the successful completion of the previous one. Progression criteria were individualized based on patient response. The RP was overseen by a co-worker (SS) at the PI’s clinic; SS was not involved in the inclusion/exclusion process at baseline. The PI had no role in subsequent patient assessments.

The goals of the acute phase (Phase I) were to: (i) prevent re-tears at the injury site, (ii) limit excessive inflammation and scar tissue formation, (iii) enhance the tensile strength, adhesion and elasticity of newly forming granulation tissue, (iv) reduce interstitial fluid accumulation and (v) identify and address any lumbo-pelvic dysfunction. Upon enrollment, patients were instructed to refrain from using medication and to follow the RICE protocol (rest, ice, compression, and elevation) to control bleeding into the muscle and limit injury extent [30].

Progression to the subacute/regeneration phase was permitted once the patient reported no pain, typically achieved within 3 to 4 days. No patient required orthopedic consultation for extended tissue damage or intramuscular hematoma, as defined by persistent pain beyond 5 days per protocol.

The goals of the subacute/regeneration phase (Phase II) included: (i) enhancing core stability, (ii) restoring strength and symmetry, (iii) alleviating pain during prone isometric hamstring contractions at 15° knee flexion, (iv) improving bilateral hamstring flexibility, (v) improving bilateral hip flexor flexibility and (vi) enhancing neuromuscular control. During this phase, patients performed daily single-session exercises involving both legs. On treatment days, exercises were completed at the PI’s clinic; on the other days, they were performed at the sports club. Exercises targeted risk factors and mechanisms contributing to hamstring injuries and were grouped into four categories: core stability and lumbopelvic control, flexibility and neural mobilization, hamstring and gluteal strength, and running technique. Aerobic conditioning began once a patient could complete three running sessions without pain or discomfort. These sessions (three per week) took place at the clinic and included four sets of 5 minutes at low to moderate intensity, self-rated by the patient. Running was suspended if pain or discomfort occurred.

Progression to the functional phase required meeting all of the following: absence of pain while prone with the knee flexed to 15°, no pain during slump test, < 10% asymmetry while in prone knee flexion at 15°, < 10% asymmetry in the active knee extension test and < 5° asymmetry in the modified Thomas Test.

The functional phase (Phase III) aimed to: (i) increase hamstring optimum length, (ii) reduce leg asymmetries in optimum length, (iii) minimize asymmetries in concentric hip extension, (iv) reduce asymmetries in horizontal force production during running and (v) enhance torsional capabilities. Daily exercises continued, with three sessions per week at the clinic and the remaining sessions at home or at the club. Exercises included core stability and lumbopelvic control, flexibility and neural mobilization, hamstring and gluteal strength, plyometric training and running technique. Running sessions during this phase involved two sets of 10 minutes at moderate to high intensity, self-rated by the patient. These sessions were suspended if pain or discomfort occurred.

Return-to-sport criteria followed established recommendations [31], and included absence of pain on palpation, flexibility tests (active knee extension and passive straight leg raise), strength tests (isometric knee flexor strength [32]) and functional tests (repeated sprint ability and single-leg bridge); comparable hamstring flexibility; psychological readiness/athlete confidence; and clearance from medical staff. The frequency and quality of supervised sessions conducted at home or the sports club were documented.

Patients in the rESWT group received the RP as described, along with up to nine rESWT sessions: three per week (at 2- to 3-day intervals), using the Swiss DolorClast device (Electro Medical Systems, Nyon, Switzerland) with the EvoBlue handpiece and a 15 mm applicator. Each session included 2500 radial extracorporeal shock waves (rESWs), with an energy flux density (EFD) of 0.12–0.16 mJ/mm^2^ (corresponding to air pressure between 3 and 4 bar, depending on patient tolerance), delivered at 15 rESWs per second (3–5 minutes per session). Treatments were performed with the patient in a prone position on an examination table, and the application site was guided by clinical and ultrasonographic findings. rESWs were applied across the entire injured muscle, from distal to proximal, and in a sagittal (dorsal–ventral) direction, without the use of local anesthesia.

**Figure 1:**
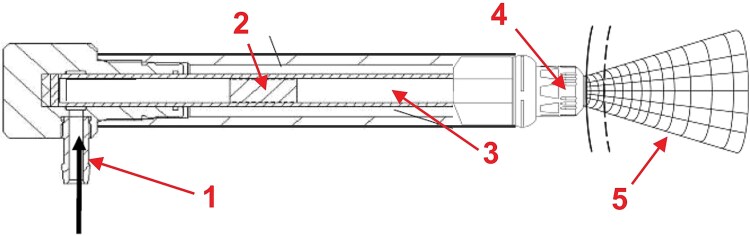
Working principle of the handpiece of a pneumatic, rESWT device (modified from [9]). Compressed air (1) is used to fire a projectile (2) within a guiding tube (3) on a metal applicator (4) that is placed on the patient’s skin. The projectile generates stress waves in the metal applicator that transmit pressure waves (rESWs) into tissue (5).

Patients in the sham rESWT group also followed the RP, receiving sham treatment with a specially designed EvoBlue handpiece that looked and sounded like the real one but emitted no rESWs. This was accomplished by mechanically blocking the projectile (‘2’ in Fig. 1) before it contacted the metal applicator (‘4’ in Fig. 1), ensuring that no shock wave energy was delivered.

### Outcome measures

The primary outcome was the individual time (in days) to return to sport, based on the criteria outlined above (according to [31]).

Secondary outcomes included individual patient satisfaction at 6 months after inclusion in the trial, assessed using a scale from 0 (maximum dissatisfaction) to 10 (maximum satisfaction), and the presence or absence of re-injury within 6 months post-inclusion. Re-injury was defined as a sudden, sharp pain in the posterior aspect of the previously injured thigh, accompanied by the same objective criteria initially used to diagnose an acute HMC injury type 3b.

### Statistical analysis

Missing data imputation was conducted in three steps: (i) Little’s MCAR test was used to assess the hypothesis that the missing data were ‘missing completely at random’ (MCAR); (ii) linear regression analysis was performed to test the hypothesis of a negative association between the durations of Phase II and Phase III of the RP; and (iii) missing data were imputed using the ‘Expectation Maximization’ (EM) technique, with a maximum of 50 iterations.

The primary outcome (individual time to return to sport) and the secondary outcome ‘patient’s satisfaction at 6 months post-inclusion into this trial’ each yielded a single data point per patient. To assess the normality of these data distributions, the D’Agostino and Pearson test was applied prior to selecting the appropriate statistical test—either the parametric Student’s *t*-test or the nonparametric Mann–Whitney test—for between-group comparisons.

The secondary outcome ‘presence or absence of re-injury during a time period of 6 months post-inclusion into this trial’ also returned a single data point (‘yes’, ‘no’, or ‘unknown’ in cases of missing data) per patient. Group comparisons for this categorical outcome were conducted using the Chi-square test.

Furthermore, group comparisons of isometric knee flexor strength post-treatment [32]—which, although not specified as an endpoint in the trial protocol [19], was evaluated as part of the return-to-sport clearance—were performed using two-way repeated measures analysis of variance (ANOVA; with injured and non-injured legs as the repeated factor), followed by uncorrected Fisher’s least significant difference (LSD) post hoc test for pairwise comparisons. The D’Agostino and Pearson test was used to assess the normality of these data distributions; however, the outcome of this test did not affect the choice of statistical analysis, as two-way repeated measures ANOVA and uncorrected Fisher’s LSD post hoc test remained appropriate. This resulted from the absence of a nonparametric equivalent for two-way repeated measures ANOVA.

All averaged data are reported as mean ± standard deviation (SD). Furthermore, a probability value of <0.05 (*P* < .05) was considered statistically significant [33]. Little’s MCAR test and the EM technique were performed using IBM SPSS Statistics (version 28.0.0.0; IBM, Armonk, NY, USA), while all other statistical analyses were conducted using GraphPad Prism (version 10.5.0 for Windows, GraphPad Software, San Diego, CA, USA).

## Results

### Compliance with trial method

This trial was conducted in accordance with the published protocol [19]. The only deviation from the protocol involved a retrospective review of all ultrasonography (US) images by an experienced investigator (PS), who had served for many years as the team physician of a German Bundesliga soccer club. This re-evaluation was performed to confirm the ultrasonographic diagnosis of acute HMC injury type 3b. Because of logistical constraints, however, this verification could only be performed after the trial had concluded. In 4 of 40 patients (10%), the ultrasonographic diagnosis of acute HMC injury type 3b could not be confirmed, as the lesion size was found to be smaller than 5 mm—resulting in a revised diagnosis of acute HMC injury type 3a. These patients were excluded from the analysis and were not replaced within the randomization scheme, yielding a modified Intent-to-Treat (mITT) population of *n* = 18 + 18 = 36.

### Baseline clinical characteristics

Table 1 presents the clinical characteristics of the patients in the mITT population at baseline (all raw data are provided in the Supplementary Information). A total of 81% (*n* = 29) of the patients were male, and the median/mean age was 26 years/25.9 ± 4.9 years, with an age range of 18 to 34 years. Regarding the type of sport, 47.2% (*n* = 17) of the patients played soccer, 27.8% (*n* = 10) played hockey, and 25% (*n* = 9) played rugby. The median/mean duration of participation in their respective sport was 11 years/12.9 ± 4.3 years (range, 6–22 years), and the median/mean number of hours of training or competition per week was 9 hours/9.8 ± 1.3 hours (range, 8–14 hours).

**Table 1:** Baseline clinical characteristics of the study patients

Characteristic	rESWT + RP (*n* = 18)	Sham rESWT + RP (*n* = 18)
Age [yr] min/median/max/mean/SD	18/24.5/34/25/4.9	18/26/34/26.7/4.8
Gender [n] male/female/non-binary	14/4/0	15/3/0
Body height [cm] min/median/max/mean/SD	151/178/194/173.7/10.9	160/179.5/187/176.9/7.4
Body weight [kg] min/median/max/mean/SD	45/72/95/69.8/14.0	49/75.5/102/75.1/12.4
BMI [kg/m^2^] min/median/max/mean/SD	19.1/23.0/27.5/22.8/2.3	19.1/23.4/29.2/23.8/2.3
Discipline [n] soccer/hockey/rugby	9/5/4	8/5/5
Years in sport min/median/max/mean/SD	6/10/22/12.4/4.4	7/13.5/20/13.4/4.2
Number of hours of sport practiced per week min/median/max/mean/SD	8/9/14/9.7/1.4	8/9.5/12/9.8/1.3
Symptoms 72 hours before enrollment no/yes	14/4	14/4
Time of event match/training	13/5	12/6
Activity at the moment of injury running/stretching	14/4	14/4
Side left/right	5/13	13/5
Injured muscle long head of biceps/semimembranosus/semitendinosus	13/3/2	12/6/0
Classification of the site of lesion (according to^29^)^a^ 1/2Acd/2Ad/2Bb/2Bcd/2 Bd/2Be/2Cc/2Cd	2/1/3/0/1/7/0/2/2	2/0/2/1/1/5/1/4/2
Size of the lesion determined during initial diagnosis [mm] min/median/max/mean/SD	5/7/15/7.9/2.6	5/7/12/8.3/2.4
Size of the lesion determined during re-examination [mm] min/median/max/mean/SD	5/9.5/15/9.4/2.8	7/9/14/9.6/2.5

^a^1, proximal musculo-tendinous junction; 2, muscle; A, proximal; B, middle; C, distal; a, intramuscular; b, myofascial; c, myofascial/perifascial; d, myotendinous; e, combined.

Of the injuries, 69.4% (*n* = 25) occurred during competition and 30.6% (*n* = 11) during training. The long head of the biceps femoris muscle was affected in 69.4% (*n* = 25) of cases, the semimembranosus muscle in 25.0% (*n* = 9) and the semitendinosus muscle in 5.6% (*n* = 2) According to a recently proposed classification system for acute muscle strain injuries developed by one of the authors (NM) [34], 11.1% (*n* = 4) of the injuries were located at the proximal musculo-tendinous junction, while 88.9% (*n* = 32) were intramuscular. Among the intramuscular injuries, 21.9% (*n* = 7) were located in the proximal part of the muscle, 59.4% (*n* = 19) in the middle portion, and 31.3% (*n* = 10) in the distal region.

The most frequently observed lesion type was myotendinous in the middle part of the muscle (33.3% of all cases; *n* = 12), followed by myofascial/perifascial in the distal region (16.7%; *n* = 6) and myotendinous in the proximal region (13.9%; *n* = 5). The median/mean lesion size, as determined by US at the time of initial diagnosis, was 7 mm/8.1 ± 2.5 mm, and 9 mm/9.5 ± 2.5 mm upon re-examination.

### Flow of patients through the trial

**Figure 2:**
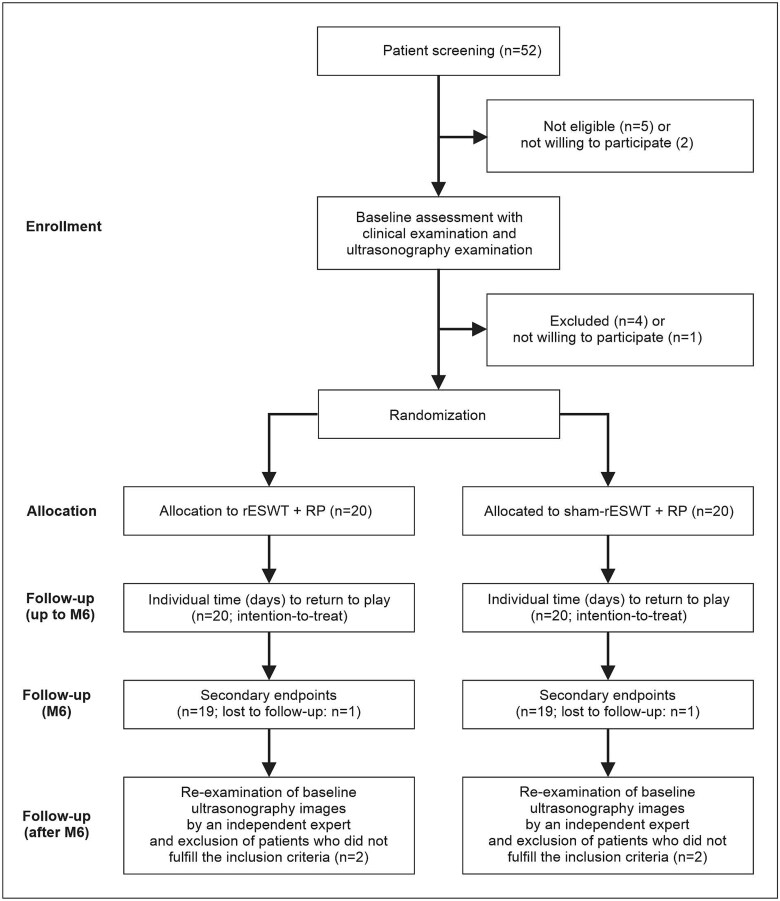
Flow of patients through the trial according to the CONSORT statement [35].

The flow of patients through the trial according to the CONSORT 2010 statement [35] is illustrated in Fig. 2. One patient in the sham rESWT + RP group was lost during Phase 3 of the RP, and one patient in the rESWT + RP group was lost during the 6-month follow-up period post-inclusion into this trial. The missing data for these patients were imputed as follows: for the patient in the sham rESWT + RP group, data on time to return to sport, satisfaction and isometric knee flexor strength were missing; for the patient in the rESWT + RP group, only satisfaction data were missing. Little’s MCAR test confirmed that the missing data were MCAR (*P* = .163).

**Figure 3:**
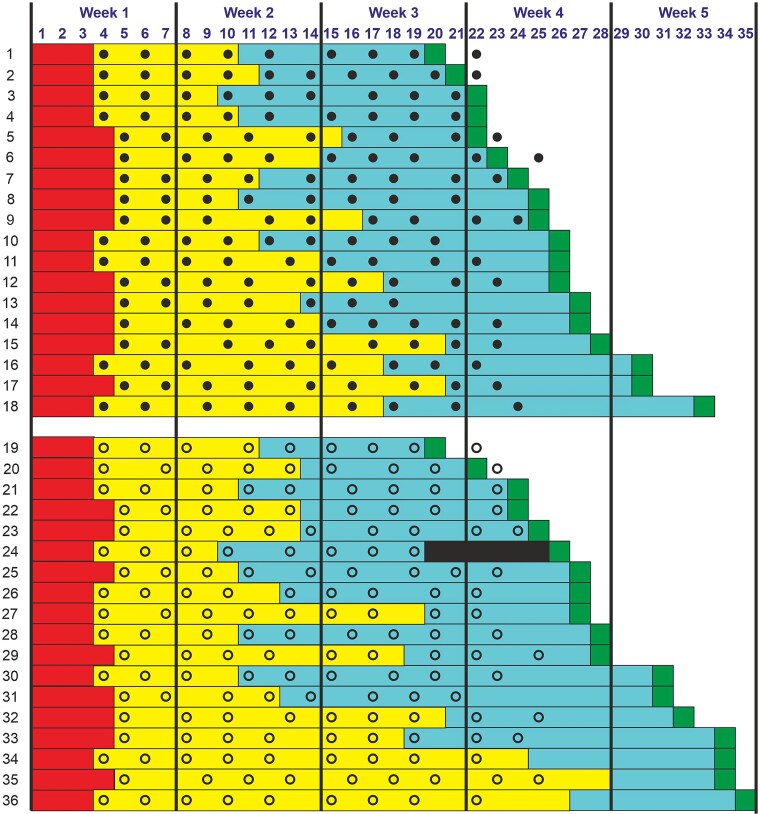
Individual time course of the patients in this trial. The horizontally arranged numbers are the days in the study (Day 1, randomization). The vertically arranged numbers represent the consecutive order of the patients according to the individual time to return to sport in the rESWT + RP group (patients 1–18) and the sham rESWT + RP group (patients 19–36) of the mITT population. For each patient the durations of Phase 1 (red), Phase 2 (yellow) and Phase 3 (blue) as well as the day of return to sport (green) are indicated, as well as all rESWT treatments (closed dots) or sham-rESWT treatments (open dots). The black rectangle in the line of patient 24 indicates the imputed missing value.

**Figure 4:**
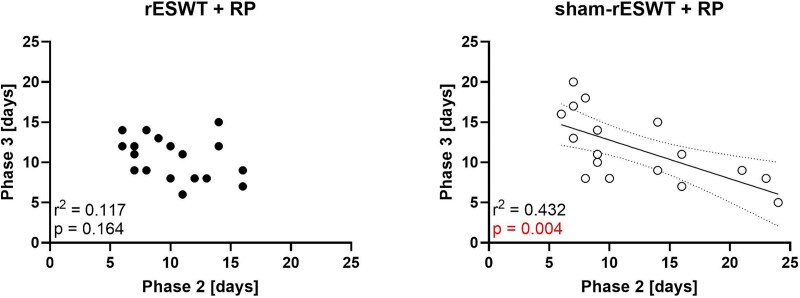
Duration of Phase 3 (functional phase) as a function of the duration of Phase 2 (subacute/regeneration phase) of all patients in this trial (except of patient 24 in the sham-rESWT + RP group who was lost during Phase III). The results of linear regression analysis are indicated.

Figure 3 illustrates the time course of all patients in the mITT population throughout the trial. In the sham rESWT + RP group, some patients exhibited a prolonged Phase II followed by a shortened Phase III (e.g. patients 35 and 36), while others demonstrated the opposite pattern—a shortened Phase II followed by an extended Phase III (e.g. patients 21 and 30). This pattern was not observed among patients in the rESWT + RP group. This observation led to the hypothesis of a negative correlation between the durations of Phase II and Phase III in the sham rESWT + RP group, which was confirmed through linear regression analysis (Fig. 4). Consequently, the missing time to return to sport for patient 24 had to be imputed based on the combined durations of Phases II and III for the patients in the sham rESWT + RP group, rather than using the duration of Phase III alone.

### Time to return to sport

The time-to-return-to-sport data for both groups in the mITT population passed the D’Agostino and Pearson test for normality (rESWT + RP group: *P* = .677; sham rESWT + RP group: *P* = .584). The median/mean time to return to sport for patients in the rESWT + RP group was 25.5 days/25.4 ± 3.5 days, and 27.5 days/28.3 ± 4.5 days for patients in the sham rESWT + RP group (see Table 2). This difference was statistically significant (unpaired, two-tailed Student’s *t*-test; *P* = .037).

**Table 2:** Results obtained for the mITT population (*n* = 36)

Outcome	rESWT + RP group (*n* = 18)	Sham rESWT + RP group (*n* = 18)	**Sign.**
Phase 1 [days] min/median/max/mean/SD	3/4/4/3.6/0.5	3/3/4/3.4/0.5	0.740^a^
Phase 2 [days] min/median/max/mean/SD	6/10/16/10.3/3.3	6/9/24/11.9/5.9	0.713^a^
Phase 3 [days] min/median/max/mean/SD	6/11/15/10.6/2.6	5/11/20/11.7/4.3	0.560^a,b^
Time to return to sport [days] min/median/max/mean/SD	20/25.5/33/25.4/3.5	20/27.5/35/28.3/4.5	0.037^c^
Satisfaction with treatment min/median/max/mean/SD	7/9/10/9.0/1.1	7/8.5/10/8.4/1.2	0.145^c^
Re-injury within 6 months after inclusion (n) no/yes/unknown	16/1/1	16/1/1	>0.999^c^

^a^result of Mann–Whitney test (*P*-value); ^b^, without the missing data of patient 24 in the sham-rESWT + RP group; ^c^, result of Student’s *t*-test (p value); ^d^, result of Chi-square test (*P*-value).

### Patient’s satisfaction

The individual patient satisfaction data for both groups in the mITT population also passed the D’Agostino and Pearson test for normality (rESWT + RP group: *P* = .276; sham rESWT + RP group: *P* = .115). The median/mean satisfaction score in the rESWT + RP group was 9/9.0 ± 1.1, and 8.5/8.4 ± 1.2 in the sham rESWT + RP group (see Table 2). This difference was not statistically significant (unpaired, two-tailed Student’s *t*-test; *P* = .145).

### Re-injury rate

In each group of the mITT population, 16 out of 18 patients (88.9%) did not experience a re-injury during the 6-month period following inclusion in the trial. Re-injury was reported in one patient per group. For one patient in each group, re-injury status could not be determined because of loss to follow-up; these missing data could not be imputed (see Table 2). The re-injury rates did not differ significantly between the groups (Chi-square test; *P* > .999).

### Isometric knee flexor strength post-treatment

The individual isometric knee flexor strength post-treatment data for both groups in the mITT population passed the D’Agostino and Pearson test for normality (previously injured leg: rESWT + RP group: *P* = .558; sham rESWT + RP group: *P* = .451; contralateral, uninjured leg: rESWT + RP group: *P* = .683; sham rESWT + RP group: *P* = .804).

**Figure 5:**
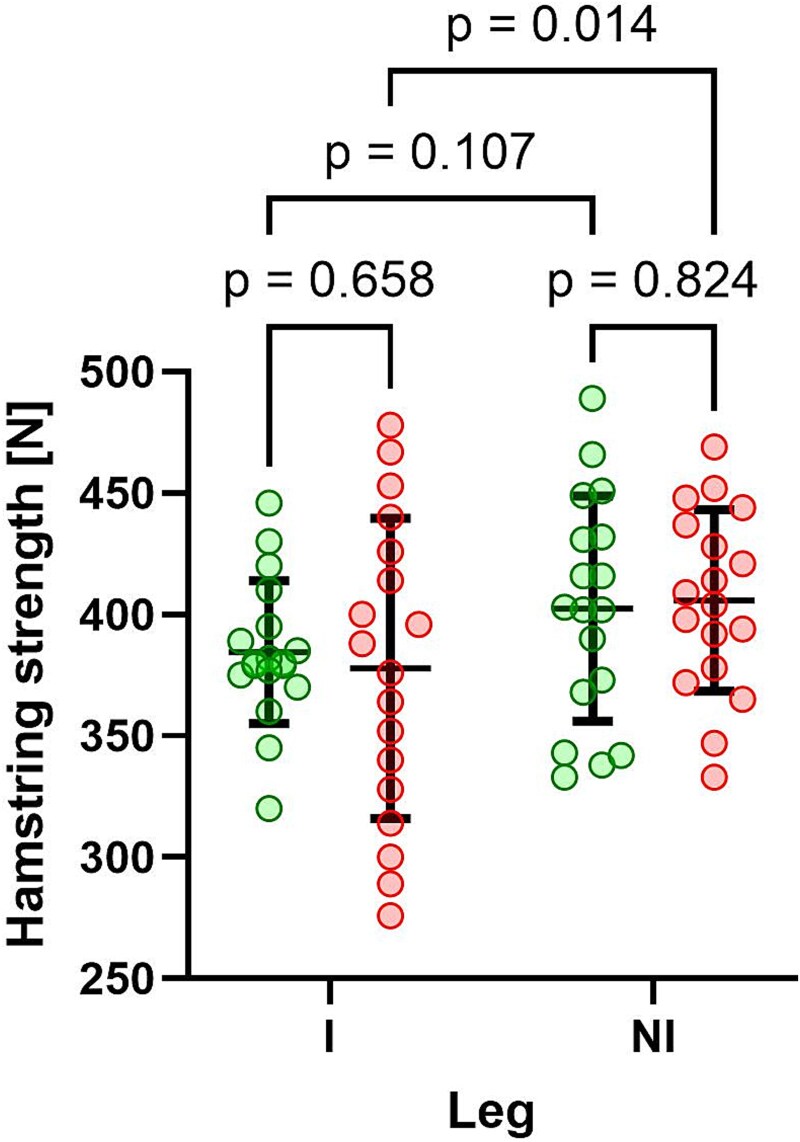
Individual data and group-specific mean ± SD of the isometric knee flexor strength post-treatment of the injured leg (I) and the non-injured leg (NI) of the patients in the rESWT + PR group (green dots) and the patients in the sham-rESWT + PR group (red dots). Results of Fisher’s LSD post hoc test for pairwise comparisons are indicated.

The lowest mean isometric knee flexor strength post-treatment was observed in the previously injured leg of the patients in the sham rESWT + RP group (378 N ± 62 N; median, 382 N), followed by the previously injured leg of the patients in the rESWT + RP group (385 N ± 29 N; median, 380 N), the contralateral, uninjured leg of the patients in the rESWT + RP group (402 N ± 29 N; median, 403 N), and the contralateral, uninjured leg of the patients in the sham rESWT + RP group (406 N ± 38 N; median, 407 N) (Fig. 5).

Two-way repeated measures ANOVA revealed a significant leg effect (previously injured vs. contralateral, uninjured; *P* = .005), but no significant treatment effect (rESWT vs. sham rESWT; *P* = .899) or interaction effect (leg × treatment; *P* = .513). Uncorrected Fisher’s LSD post hoc testing showed a significant difference between the previously injured and contralateral, uninjured leg of the patients in the sham rESWT + RP group (*P* = .014); all other pairwise comparisons were not statistically significant (Fig. 5).

### Safety of the interventions

No serious adverse events occurred during this trial.

## Discussion

The first key finding of this study was that the specific RP used resulted in a median time to return to sport after acute HMC injury type 3b (sham rESWT + RP group: 27.5 days), which was shorter than corresponding time intervals reported by (i) Ekstrand *et al.* [18] for elite soccer players with moderate partial muscle tears (type 3b) of the posterior thigh muscles: 30 days (mean ± SD: 35.5 ± 19.5 days), (ii) Reurink *et al.* [8] for competitive and recreational athletes (median time to return to sport after injection of platelet rich plasma (PRP) or isotonic saline as placebo for hamstring injuries diagnosed on MRI, defined by increased signal intensity on STIR and/or T2-weighted images localized to a single region within the muscle): 42 days, and (iii) Bayer *et al.* [26] for amateur athletes with acute thigh injuries (60% of patients) or calf injuries (40%) confirmed via US and MRI: 62.5 days with early therapy initiated on day 2 post-injury or 83 days with delayed therapy starting on day 9 post-injury. Additionally, the range of return-to-sport times in the sham rESWT + RP group (20–35 days) differed from the range reported for type 3b muscle injuries in the guidelines of the Italian Society of Muscles, Ligaments and Tendons (25–35 days) [6]. These discrepancies may be attributed to variations in patient selection, specific treatment protocols, definitions of return to sport or complex interactions among these and other contributing factors. Regardless, the RP used in this study appears promising and may serve as a suitable control therapy in future studies on muscle injury treatment.

The second key finding was that the addition of rESWT to the RP further reduced the median/mean time to return to sport by 2/2.9 days, corresponding to a relative reduction of 7.3%/10.3%. Although this improvement may appear modest at first glance, it holds particular relevance in professional sports, where even a single day of lost player activity can result in substantial financial costs. For instance, assuming that a professional athlete earns a gross annual salary of US$250 000, a reduction in return-to-sport time by 3 days translates into cost savings exceeding US$2000—sufficient to justify the cost of additional rESWT sessions. The Global Sports Salaries Survey 2019 [36] provides context for this estimate: in 2019, the average annual salary for professional soccer players was US$1.4 million in France’s Ligue 1, US$2.1 million in the German Bundesliga, US$2.4 million in Italy’s Serie A, US$2.7 million in Spain’s La Liga, and US$4.2 million in the English Premier League (all calculated using an exchange rate of 1 GBP = US$1.326 as of 31 December 2019). In none of these leagues did the average annual salary fall below US$250 000 [36]. Similarly, in the United States National Basketball Association, the average annual salary for first-team players in 2019 ranged from US$7.1 million (New York Knicks) to US$10.0 million (Portland Trail Blazers) [36].

Importantly, the observed reduction in mean return-to-sport time with rESWT was not associated with any adverse events, including re-injury. This is particularly noteworthy given prior concerns that early mechanical stress on injured muscle tissue may increase the risk of heterotopic ossification (HO) and myositis ossificans following significant soft tissue trauma [3]. However, rESWT does not appear to exert this detrimental effect. On the contrary, early application of rESWT may inhibit molecular pathways responsible for recruiting or differentiating circulating cells that contribute to HO, thereby potentially preventing the transition of pre-osseous tissue into mature bone [37]. Once HO is established, ESWT may alleviate clinical symptoms but cannot reverse the ossification [38, 39].

An unexpected finding of this study was the observation that, following sham rESWT + RP, the mean isometric knee flexor strength post-treatment in the previously injured leg was slightly—but significantly—lower than that of the contralateral, uninjured leg, whereas no such difference was observed after rESWT + RP. This was particularly notable given that the mean time to return to sport was significantly shorter in the rESWT + RP group compared to the sham rESWT + RP group. None of the previously cited studies by Ekstrand *et al.* [18], Reurink *et al.* [8] or Bayer *et al.* [26] reported post-treatment muscle strength comparisons between the previously injured and contralateral uninjured sides.

Among the five clinical studies on ESWT for muscle injuries included in a recent systematic review [16], only two assessed muscle strength. One of these was a RCT investigating the effect of ESWT on experimentally induced delayed-onset muscle soreness (type 1b) of the non-dominant elbow flexors in healthy subjects [40], which is not comparable to the treatment of structural type 3b muscle injuries examined in the present study. The other was a case series involving seven amateur athletes with grade II muscle injuries and one athlete with a grade III muscle injury (further classification details of the muscle injuries not provided) [41]. These patients, all with muscle injuries in the legs of >3 weeks’ duration, received physiotherapy in addition to two sessions of ESWT (the first on the day after enrollment and the second 3 weeks later), with a reported mean return-to-sport time of 8.75 weeks (range, 4–16 weeks). Although all patients demonstrated muscle strength grade V at 8 weeks post-enrollment, no comparison was made between the injured and contralateral, uninjured limbs. Given the substantial differences in study design—particularly the post-injury timing of enrollment and the ESWT protocol—this study [41] cannot be directly compared with the present investigation. The remaining three studies included in that systematic review [16] did not assess post-treatment muscle strength. These included a case series on professional soccer players [15], a case report involving a grade III tear of the plantaris muscle with associated hematoma [42], and a case report of a previously healthy adult with a traumatic hematoma in the left calf [43].

In summary, this study is the first to report the restoration of muscle strength following the treatment of structural muscle injuries using rESWT + RP—a result that was not achieved with sham rESWT + RP. In this context, rESWT was demonstrated to enhance both the structural and functional recovery of muscle tissue following type 3b structural injury.

Basic science and animal model studies provide mechanistic support for the therapeutic efficacy of rESWT in acute structural muscle injuries. One proposed mechanism is the direct stimulation of resident muscle cells to regenerate damaged tissue. In a rat model of experimentally induced acute type 3 muscle tears, treatment with rESWs (generated using the same rESWT device employed in the current study) resulted in fewer mononucleated cells and a higher number of newly formed muscle fibers at 4 and 7 days post-treatment, compared to both diclofenac-treated and untreated controls [14]. Additionally, rats treated with rESWT showed increased RNA expression of MyoD and Myosin, markers of muscle regeneration [14]. Similar findings were reported in cultured human skeletal muscle cells exposed to rESWs generated with the same device, including increased cell viability and upregulation of muscle-specific genes such as Myf5, MyoD, Pax7, and NCAM [13].

Although ESWT enhances pro-angiogenic gene expression in skeletal muscle, it does not appear to increase the actual density of blood vessels in treated muscle tissue [14, 44]. Despite early reports showing ESWT-induced upregulation of angiogenesis-related growth factors such as eNOS (endothelial nitric oxide synthase), VEGF (vascular endothelial growth factor) and PCNA (proliferating cell nuclear antigen) [45], the commonly cited effect of ESWT in promoting neoangiogenesis appears more relevant in tissues such as the tendon–bone junction (focused ESWT) [46] and skin (rESWT) [47], rather than in skeletal muscle regeneration.

Beyond cellular regeneration, rESWT may aid recovery through additional mechanisms. For instance, injured muscles often develop increased tone, which can hinder healing and is sometimes treated with local anesthetics [3]. However, intramuscular injection of anesthetics typically induces reversible myonecrosis, with regeneration taking three to 4 weeks [48]. In contrast, rESWT offers a non-invasive means of reducing muscle tone without impairing function and is already in use for treating spasticity [49–51]. Kenmoku *et al.* [52] further demonstrated a dose-dependent reduction in mean compound muscle action potential (CMAP) following rESW application in rat muscle tissue (in all cited studies [49–52], rESWs were generated using the same device employed in the current study).

As for rESWT treatment parameters for acute HMC injury type 3b—including frequency of treatment sessions, number of rESWs per treatment session, EFD and rESW frequency—the current study protocol offers a useful baseline. However, fixed parameters are less practical in clinical settings than in controlled research environments. In this context, a retrospective case series used a highly individualized rESWT protocol for treating acute muscle injuries (types 1a, 2b and 3a) in professional soccer players [15]. In that study, the EFD was adjusted individually, allowing patients to experience mild discomfort without pain. The number of rESWs per session ranged from 6000 to 12 000, and treatments were administered daily or every other day. Future protocols—both clinical and research—should consider similarly individualized parameters to account for variability in patient response, injury severity and other relevant factors.

### Limitations

This study has three main limitations. First, establishing definitive evidence that the combination of rESWT and the specific RP used in this study is superior to the RP alone in reducing return-to-sport times and restore muscle strength will require investigation in a larger cohort of injured athletes. Specifically, based on the return-to-sport times reported in this study [rESWT + RP group: 25.4 ± 3.5 days (mean ± SD); sham rESWT + RP group: 28.3 ± 4.5 days], a total of at least 2 × 31 = 62 (or 2 × 41 = 82) patients would need to be enrolled in a RCT to demonstrate superiority of rESWT + RP over sham rESWT + RP with a two-sided confidence level of 95% and a statistical power of 80% (or 90%). These calculations were performed using the Open Source Epidemiologic Statistics for Public Health software [53]. The relatively small sample size originally calculated in the protocol of this study [19] was based on the prior experience of two authors (PS and CS) in treating elite athletes with HMC injury type 3b using rESWT, including a case involving a professional soccer player from a European top-tier club (competing in both the UEFA Champions League and the FIFA World Cup), who sustained a type 3b injury and returned to full competitive play (90-minute match) within 35 days. This was in contrast to the findings of two earlier studies [8, 26], which reported cumulative probabilities of return to sport on day 35 post-injury of only 20% [8] or 5% [26] after treatment with RPs alone. However, it became evident that the specific RP employed in the current study led to substantially shorter mean and median return-to-sport times compared to the RPs used in those earlier investigations [8, 26].

Second, the diagnosis of acute HMC injury type 3b in this study was made using US rather than magnetic resonance imaging (MRI). This decision was driven by economic considerations, as the cost of MRI scans for muscle injuries in Argentina is disproportionately high relative to the athletes’ salaries (with the exception of some professional athletes). Nonetheless, both US and MRI are effective modalities for diagnosing hamstring strains and tendinopathy [6,54–56], and both provide detailed information regarding the localization and characterization of HMC injuries [54].

Third, 10% of the patients enrolled in this RCT were retrospectively excluded because the ultrasonographic diagnosis of acute HMC injury type 3b could not be confirmed by an experienced investigator (PS), who had served for many years as the team physician of a German Bundesliga soccer club. Ideally, this independent review of ultrasound scans should have been conducted prior to patient inclusion; however, logistical constraints made this infeasible. Including the data of all 40 patients initially enrolled (provided in the Supplementary Information) would have yielded the following outcomes: minimum/median/maximum/mean ± SD return-to-sport times of 20/26/33/25.8 ± 3.5 days in the rESWT + RP group, and 20/27.5/35/28.2 ± 4.7 days in the sham rESWT + RP group, with a p value of 0.069 (unpaired, two-tailed Student’s *t*-test). However, this would effectively constitute a different study—one examining patients with both type 3a and 3b acute HMC injuries—which was not the intended scope of this investigation. Therefore, reporting the results from the mITT population as defined in this study is justified.

A minor limitation of this study is that the evaluation of isometric knee flexor strength post-treatment did not take into account whether the previously injured leg was the dominant or non-dominant one. However, this distinction may be relevant primarily for soccer players (who comprised 47.2% of the athletes enrolled in this study), but not necessarily for hockey and rugby players (who together accounted for 52.8% of the study population). A recent systematic review and meta-analysis found that, in soccer players, hamstring injuries are only 1.25 times more likely to occur in the dominant leg [57], suggesting that the pathomechanisms of acute HMC injuries are not primarily related to kicking a ball. Kicking predominantly involves hip flexor (iliopsoas, rectus femoris, sartorius, tensor fasciae latae) and knee extensor muscles (quadriceps femoris group). Instead, anatomical and functional characteristics of the HMC itself appear to predispose it to injury—specifically, the biarticular nature of the muscles and their engagement in eccentric contraction during gait and running [25]. Acute HMC injuries typically occur via an eccentric mechanism during the terminal phase of the swing cycle in running [58]. In summary, the fact that the evaluation of isometric knee flexor strength post-treatment did not account for whether the previously injured leg was the dominant or non-dominant one may be considered negligible.

## Conclusion

In the rehabilitation of athletes with acute HMC injury type 3b, where every day until return to sport is critical, the combination of rESWT and the specific RP applied in this study is more effective than the RP alone, and the latter is superior to other RPs described in the literature.

## Supplementary Material

Supplementary_Data_ldaf009

## Data Availability

Relevant raw data are provided in Supplementary Information.
