# Supplementary material for: Radial ESWT combined with a specific rehabilitation program (rESWT+RP) is more effective than sham rESWT+RP for acute hamstring muscle complex injury type 3b: a randomized, controlled trial
Source: Br Med Bull. 2025 Sep 2;155(1):ldaf009. doi: 10.1093/bmb/ldaf009 (PMC12403062; doi:10.1093/bmb/ldaf009)
Supplement: Supplementary_Data_ldaf009 [file supplementary_data_ldaf009.docx]

**Radial ESWT combined with a specific rehabilitation program (rESWT + RP) is more effective than sham rESWT + RP for acute hamstring muscle complex injury type 3b: a randomised, controlled trial**

Javier Crupnik, Santiago Silveti, Natalia Wajnstein, Alejandro Rolon, Tobias Wuerfel,

Peter Stiller, Antoni Morral, John P. Furia, Nicola Maffulli, Christoph Schmitz

**Supplementary Data**

This supplement contains additional information about the following variables of the patients in this trial.

| **Variable** | **Description** |
| --- | --- |
| V1 | Treatment in addition to the specific rehabilitation program |
| V2 | Activity at the moment of injury (running / stretching) |
| V3 | Injured muscle (long head of biceps / semimembranosus / semitendinosus |
| V4 | Classification of the site of lesion**^^[[1]](#footnote-1)^^** |
| V5 | Size of the lesion determined during initial diagnosis [mm] |
| V6 | Size of the lesion determined by retrospective re-assessment [mm] |
| V7 | Duration of Phase I [days] |
| V8 | Duration of Phase II [days] |
| V9 | Duration of Phase III [days] |
| V10 | Time to return to sport [days] |

| **No^[[2]](#footnote-2)^** | **V1** | **V2** | **V3** | **V4^^[[3]](#footnote-3)^^** | **V5** | **V6** | **V7** | **V8** | **V9** | **V10** |
| --- | --- | --- | --- | --- | --- | --- | --- | --- | --- | --- |
| 1 | rESWT | running | long head of biceps | 2,B,d | 6 | 7 | 3 | 7 | 9 | 20 |
| 2 | rESWT | stretching | semimembranosus | 1,d/c | 15 | 15 | 3 | 8 | 9 | 21 |
| 3 | rESWT | running | semimembranosus | 2,B,d | 6 | 6 | 3 | 6 | 12 | 22 |
| 4 | rESWT | running | long head of biceps | 2,C,d | 5 | 5 | 3 | 7 | 11 | 22 |
| 5 | rESWT | running | long head of biceps | 2,B,d | 7 | 10 | 4 | 11 | 6 | 22 |
| 6 | rESWT | running | long head of biceps | 2,C,d | 7 | 10 | 4 | 10 | 8 | 23 |
| 7 | rESWT | stretching | long head of biceps | 2,B,d | 5 | 6 | 4 | 7 | 12 | 24 |
| 8 | rESWT | running | long head of biceps | 2,A,d | 6 | 7 | 4 | 6 | 14 | 25 |
| 9 | rESWT | running | long head of biceps | 2,B,d | 7 | 9 | 4 | 12 | 8 | 25 |
| 10 | rESWT | running | long head of biceps | 2,C,b | 6 | 9 | 3 | 8 | 14 | 26 |
| 11 | rESWT | running | long head of biceps | 2,A,d | 8 | 8 | 3 | 11 | 11 | 26 |
| 12 | rESWT | running | long head of biceps | 2,C,c | 10 | 11 | 4 | 13 | 8 | 26 |
| 13 | rESWT | running | long head of biceps | 2,B,d | 7 | 13 | 4 | 9 | 13 | 27 |
| 14 | rESWT | stretching | semimembranosus | 2,B,d/c | 7 | 9 | 4 | 10 | 12 | 27 |
| 15 | rESWT | stretching | semitendinosus | 1,c/d | 10 | 10 | 4 | 16 | 7 | 28 |
| 16 | rESWT | running | long head of biceps | 2,A,d | 12 | 15 | 3 | 14 | 12 | 30 |
| 17 | rESWT | running | long head of biceps | 2,B,d | 10 | 10 | 4 | 16 | 9 | 30 |
| 18 | rESWT | running | semitendinosus | 2,A,d/c | 9 | 10 | 3 | 14 | 15 | 33 |
| 19 | sham rESWT | running | semimembranosus | 2,A,d | 9 | 12 | 3 | 8 | 8 | 20 |
| 20 | sham rESWT | running | long head of biceps | 2,B,e | 9 | 12 | 3 | 10 | 8 | 22 |
| 21 | sham rESWT | running | semimembranosus | 2,B,d | 7 | 10 | 3 | 7 | 13 | 24 |
| 22 | sham rESWT | stretching | semimembranosus | 2,C,c | 7 | 7 | 4 | 9 | 10 | 24 |
| 23 | sham rESWT | running | long head of biceps | 2,C,c | 7 | 7 | 4 | 9 | 11 | 25 |
| 24 | sham rESWT | stretching | semimembranosus | 2,B,d | 12 | 12 | 3 | 6 | 16**^[[4]](#footnote-4)^** | 26 |
| 25 | sham rESWT | running | long head of biceps | 2,B,d | 5 | 8 | 4 | 6 | 16 | 27 |
| 26 | sham rESWT | running | long head of biceps | 2,C,d | 6 | 8 | 3 | 9 | 14 | 27 |
| 27 | sham rESWT | running | long head of biceps | 2,B,d | 9 | 9 | 3 | 16 | 7 | 27 |
| 28 | sham rESWT | running | long head of biceps | 2,C,c | 7 | 9 | 3 | 7 | 17 | 28 |
| 29 | sham rESWT | running | long head of biceps | 1,d | 12 | 12 | 4 | 14 | 9 | 28 |
| 30 | sham rESWT | running | long head of biceps | 2,B,d | 7 | 8 | 3 | 7 | 20 | 31 |
| 31 | sham rESWT | running | long head of biceps | 2,A,d | 12 | 12 | 4 | 8 | 18 | 31 |
| 32 | sham rESWT | running | long head of biceps | 1,d/c | 12 | 14 | 4 | 16 | 11 | 32 |
| 33 | sham rESWT | stretching | semimembranosus | 2,B,d/c | 10 | 10 | 4 | 14 | 15 | 34 |
| 34 | sham rESWT | running | long head of biceps | 2,C,c | 6 | 8 | 3 | 21 | 9 | 34 |
| 35 | sham rESWT | stretching | long head of biceps | 2,C,d | 5 | 7 | 4 | 24 | 5 | 34 |
| 36 | sham rESWT | running | semimembranosus | 2,B,b | 7 | 7 | 3 | 23 | 8 | 35 |
| **^[[5]](#footnote-5)^** | rESWT | running | long head of biceps | 2,B,b | 7 | <5 | 4 | 16 | 7 | 28 |
| **^5^** | rESWT | running | semitendinosus | 2,B,d | 7 | <5 | 3 | 14 | 12 | 30 |
| **^5^** | sham rESWT | running | long head of biceps | 2,A,d | 5 | <5 | 3 | 24 | 6 | 34 |
| **^5^** | sham rESWT | running | semimembranosus | 2,B,d | 6 | <5 | 4 | 13 | 3 | 21 |

1. Classification according to Chan O, Del Buono A, Best TM, Maffulli N. Acute muscle strain injuries: a proposed new classification system. *Knee Surg Sports Traumatol Arthrosc* 2012;20:2356-62. doi: 10.1007/s00167-012-2118-z. [↑](#footnote-ref-1)
2. These numbers represent the consecutive order of the patients according to the invidivual time to return to sport in the rESWT + RP group (patients 1-18) and the sham rESWT + RP group (patients 19-36) in the mITT population. They are identical to the corresponding numbers in Figure 3 in the manuscript. [↑](#footnote-ref-2)
3. Abbreviations: 1, proximal musculo-tendinous junction; 2, muscle; A, proximal; B, middle; C, distal; a, intramuscular; b, myofascial; c, myofascial/perifascial; d, myotendinous; e, combined. [↑](#footnote-ref-3)
4. Missing data imputed using the using the *Expectation Maximization Technique*.  [↑](#footnote-ref-4)
5. These patients were retrospectively excluded because the ultrasonographic diagnosis of acute HMC injury type 3b could not be confirmed by an experienced investigator (PS) who served as team physician of a German Bundesliga soccer club for many years. [↑](#footnote-ref-5)
